# In Ischemic Heart Disease, Reduced Sensitivity to Pressure at the Sternum Accompanies Lower Mortality after Five Years: Evidence from a Randomized Controlled Trial

**DOI:** 10.3390/jcm12247585

**Published:** 2023-12-08

**Authors:** Søren Ballegaard, Jens Faber, Christian Selmer, Finn Gyntelberg, Svend Kreiner, Benny Karpatschof, Tobias Wirenfeldt Klausen, Åke Hjalmarson, Albert Gjedde

**Affiliations:** 1 Endocrine Unit, Department of Medicine, Herlev-Gentofte University Hospitals, 2730 Herlev, Denmark; jens.faber@regionh.dk (J.F.);; 2 Faculty of Health and Medical Sciences, University of Copenhagen, 2200 Copenhagen, Denmark; 3 Department of Endocrinology, Bispebjerg-Frederiksberg University Hospitals, 2400 Copenhagen, Denmark; 4 The National Research Center for the Working Environment, 2100 Copenhagen, Denmark; 5 Institute of Biostatistics, University of Copenhagen, 1017 Copenhagen, Denmark; 6 Institute of Psychology, University of Copenhagen, 1017 Copenhagen, Denmark; 7 Department of Cardiology, Sahlgrenska University Hospital, University of Gothenburg, 41345 Gothenburg, Sweden; 8 Department of Neuroscience, University of Copenhagen, 2200 Copenhagen, Denmark; 9 Translational Neuropsychiatry Unit, Department of Clinical Medicine, Aarhus University, 8000 Aarhus, Denmark; 10 Department of Neurology and Neurosurgery, McGill University, Montreal, QC H3A 2B4, Canada

**Keywords:** autonomic nervous system, sympathetic hyperactivity, autonomic nervous system dysfunction, ischemic heart disease, mortality, periosteal pressure sensitivity

## Abstract

**Background:** Autonomic nervous system dysfunction (ANSD) is associated with negative prognosis of ischemic heart disease (IHD). Elevated periosteal pressure sensitivity (PPS) at the sternum relates to ANSD and sympathetic hyperactivity. Two previous observational case–control studies of the effect of reduction of PPS suggested lower all-cause mortality from IHD and stroke. We now used a specific daily, adjunct, non-pharmacological program of reduction of elevated PPS to test the hypothetical association between the intervention and reduced all-cause mortality in patients with stable IHD in a randomized controlled trial (RCT). **Methods:** We completed active (n = 106) and passive interventions (n = 107) and compared the five-year mortalities. We also compared the five-year individual all-cause mortality of each participant to approximately 35.000 members of the general population of Denmark. Pooling the mortality data from the active group of the RCT with the two preliminary studies, we registered the mortality following active intervention of 1.168 person-years, compared to 40 million person-years of the pooled general population. **Results:** We recorded fewer deaths of the active RCT intervention group than of the corresponding control group from the general population (*p* = 0.01), as well as of the passive RCT intervention group (*p* = 0.035). The meta-analysis of the three studies together demonstrated reduced 4.2-year all-cause mortality of 60% (*p* = 0.007). **Conclusions:** The test of the hypothetical effect of an intervention aimed at the attenuation of ANSD accompanied by a lowered PPS revealed reduced all-cause mortality in patients with stable IHD.

## 1. Introduction

Ischemic heart disease (IHD) is a leading cause of death according to the Global Burden of Disease study [1]. A wide range of physiological, biochemical, and psychological variables, all controlled by the autonomic nervous system (ANS), have been identified as cardiovascular risk factors [2,3]. These factors include heart rate [4], work of the heart measured as the pressure rate product (i.e., the product of systolic blood pressure and heart rate) [5], the metabolic syndrome [6], chronic low-grade inflammation [7], persistent stress [8], and depression [9], all associated with ANS dysfunction (ANSD) [10].

The ANS regulates functions of the human body that maintain the stable interior environment in the face of changing exterior conditions. The stable state reflects a mechanism of resilience known as homeostasis [11], reached by constant adjustment of a dynamic balance between the sympathetic and parasympathetic nervous systems [12,13]. ANSD reflects a failure of this resilience that may appear as sympathetic hyperactivity or predominance [14], associated with morphological and functional changes diagnosed by multiple tests that include heart rate variability [14] and table tilting [15]. Current treatments address the symptoms but not the possible autonomic dysfunction itself [14], with little conclusive evidence of any effective rectification of ANSD.

In myocardial infarction and unstable angina pectoris, ANSD shows as prolonged excessive sympathetic activity [16]. Peripheral nerve stimulation with hypothetical modulation of central ANS in principle would serve as a novel therapeutic approach to IHD management, but there are no reports of the potential efficacy of a special focus on the reduction of ANSD. Neuromodulation using non-noxious cutaneous sensory nervous stimulation is a well-known and widely used treatment for newborn infants, known as skin-to-skin treatment or Kangaroo Mother Care. In premature neonates, neuromodulation positively affected brain development with substantial increase of overall survival [17]. Non-noxious cutaneous sensory nerve stimulations contributed to these effects by reducing autonomic sympathetic activity through the release of oxytocin from the hypothalamus [18].

Periosteal pressure sensitivity (PPS) has been shown to be a measure of ANS function, and elevated PPS reflects ANSD [19]. We completed two prospective, long-term case–control studies of 103 patients with IHD followed for 3 years, and of 73 stroke patients followed for 4.5 years, using a non-pharmacological cardiac rehabilitation program of daily measurements of PPS as indicators of ANS function, and ad hoc non-noxious cutaneous sensory nerve stimulation claimed to reduce excess sympathetic activity, recorded as reductions of elevated PPS values. As adherence to traditional non-pharmacological cardiac rehabilitation programs is low [20,21], the present PPS-guided cardiac rehabilitation program encouraged compliance.

The studies revealed reduced all-cause mortality using this intervention, compared to the general Danish population [22,23], and formed the basis of the hypothesis tested in this study: a specific, adjunct, non-pharmacological educational program, using daily PPS recordings and non-noxious cutaneous sensory nerve stimulation, by means of a hypothetical alleviation of ANSD, would lower five-year all-cause mortality in persons with IHD. To test the hypothesis, we completed this observer-blinded randomized controlled trial (RCT) with intention-to-treat (ITT) analysis to ensure randomization with respect to unknown confounding factors and to exclude potential selection and researcher bias of the previous case–control studies.

We now report the five-year all-cause mortality of the group of the active intervention, defined as conventional cardiac rehabilitation plus three months of education of participants aimed at a reduction in elevated PPS measures over time and ad hoc treatment of angina pectoris attacks, focused on persistently improved empowerment and compliance. We compared the active intervention to the group of passive intervention who received conventional cardiac rehabilitation only. We compared the two groups with corresponding control groups from the general Danish population, matched for gender, sex, and observation period. Further, we completed this study with a combined meta-analysis of all three studies using the same educational program.

## 2. Materials and Methods

### 2.1. Ethics

All participants provided written informed consent after oral and written information about this study. This study was performed in accordance with the declaration of Helsinki. This study was approved by the local ethics committee (Regional Ethics Committee of the Copenhagen Region, Kongensvænge 2, DK-3400 Hillerød, www.regionh.dk/vek, identifier H-4-2010-135 (date: 12 January 2012), and amendment 31962 (date: 12 April 2012)) and by the Danish Data Protection Agency (identifier 2011-41-7022), registered on www.clinicaltrials.gov (identifier NCT01513824) (date: 17 January 2012).

### 2.2. Design and Participants

**Figure 1:**
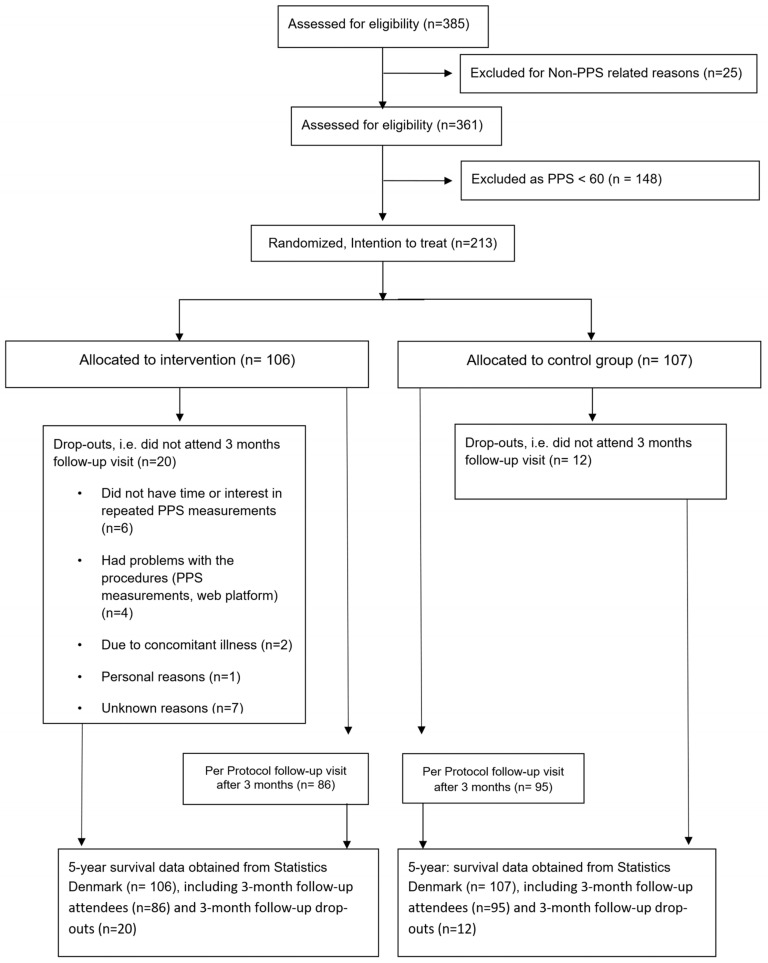
CONSORT diagram [24,25].

The study population comprises a cohort of 361 consecutively diagnosed patients with stable IHD recruited in 2011, of whom 65% had elevated PPS measures (defined as equal to or greater than 60 arbitrary units (defined by logistic transformation of the applied pressure in units of kPa) and indicative of chronic stress based on receiver operation characteristic curve analysis for specificity and sensitivity with respect to depression [24,25] The intervention groups included the two groups comprising an active or passive intervention. As shown in Figure 1, we randomly assigned 213 patients with stable documented IHD, defined as a history of myocardial infarction, percutaneous intervention, or coronary artery bypass grafting, having completed cardiac rehabilitation more than six months prior to inclusion. All participants had elevated PPS, with randomization 1:1 to the group of the active intervention for three months, as described below (n = 106), or to the group of the passive intervention (n = 107).

All participants received written information that we regarded the level of persistent stress as elevated, and that persistent stress has a negative effect on heart disease. The calendar years of enrollment into this study included 2011 and 2012 for 80 and 26 patients of the active intervention group and for 79 and 28 patients of the passive intervention group, respectively. The period of study ended 31 December 2017. Researchers and patients had no further contact after the first three months that defined the months of contact as periods of an active or passive intervention, depending on the group, with no further contact during the remaining 5 years.

At the end of three months of the active or passive intervention, the patients of the cohort underwent two randomized clinical trials (RCT). The trials tested the response of the two groups of participants to the active versus passive intervention in terms of changes to periosteal pressure sensitivity (PPS) after three months [24,25,26]. The first trial tested the hypothesis that the active intervention for three months would not influence depression scores when compared to the passive intervention [25]. The second trial tested the hypothesis that changes of PPS would not reflect the changes of systolic blood pressure and heart rate in response to tilt table testing after three months of the active versus passive intervention [26]. Both hypotheses were rejected by the respective tests.

We limited the follow-up period in the original protocol to 5 years, consistent with the longest period of uninterrupted accessibility to mortality data from the general Danish population by Statistics Denmark. We compared the five-year all-cause survival of each of the 106 persons of the active intervention RCT group with approximately 35,000 individuals matched for age, gender, and observation period. We created a control group from the general population, based on approximately 19,000,000 person-years, calculated as 106 participants times the 35,000 corresponding persons in the general population for each participant times the 5 years of observation. The corresponding number of person-years in the active RCT group was 530 person-years, calculated as 106 participants times 5 years of observation, with a similar calculation made for the passive RCT group.

### 2.3. Outcome Measures

We separately compared the five-year all-cause mortality of the two groups of the RCT study. Using a unique identification code, Statistics Denmark cross-linked anonymized data on all individuals living in Denmark in terms of mortality, from which register we obtained the outcome measure in terms of all-cause mortality. Due to local data regulations, we presented numbers of clinical events of less than or equal to three in the statistical analysis as “three or fewer events”.

### 2.4. PPS Measurement

The PPS device measures the sensitivity of the polymodal sensory nervous system to periosteal pressure at the most sensitive point on the sternum between costae 3 and 5, representing a specific area of segmental innervation of the heart [27], identified by finger pressure. After 10 min of rest in the supine position, the participant first learned the technique, becoming familiar with the procedure with two measurements at a control point on the dorsal part of the middle phalanx of the left index finger. The study nurse manually applies the instrument with gradually increasing pressure until the participant says stop when the threshold between discomfort and pain is reached: in total, allowing as many as five seconds of pressure duration. A withdrawal reflex, typically the startle reflex from the eyes with an eye blink, is considered a stop signal. The procedure is then repeated at the most tender place of the sternum, identified via a palpation by the observer. The procedure at the sternum is conducted twice, with the PPS measure adopted as the mean of the two recordings. However, when the two recordings differ by more than 10 PPS units, a third measurement is conducted, and the PPS measure is calculated as the mean of the three recordings. The instrument displays a number on a scale from 30 to 100 (indicative of pressure sensitivity thresholds from 400 to 25 kilopascals, i.e., a factor of 16), where increasing sensitivity is indicated by increasing numbers. The high PPS measure reflects a high level of autonomic dysfunction (high sensitivity, low pain threshold).

Participants conducted daily home recordings of PPS. A website was established for this study (www.songheart.org), and each participant in the active group received a personal profile with login details with the option to enter the PPS measures for personal track recording and to make ongoing professional surveillance possible, and thus with proactive contact in case of deviating or missing PPS measurements. To minimize bias, the measurements are invisible to both the instructor and participant until the end of the measurements.

### 2.5. Interventions

All participants completed cardiac rehabilitation more than six months prior to inclusion. Upon inclusion into the RCT, all participants, active as well as passive intervention group members received the information that the level of persistent stress was elevated, as a sign of poor cardiovascular health. Both groups received an 80-page manual of general stress management from the perspective that persistent stress negatively affects IHD. All medication of active and passive group members remained unchanged during the last month prior to the baseline examination, and all participants received instructions not to change medication during the initial 3-month period of participation. Thereafter, medication was administered by the general practitioner. The interventions included no new medication (Table 1).

### 2.6. Active Intervention

Active intervention group members underwent a specific 3-month educational program of non-pharmacological self-care with assignments from a personal instructor. The education had two elements, a preventive part aimed at the reduction of elevated sympathetic activity (ANSD), believed to be measurable as elevated resting PPS, and an active intervention part aimed at an ad hoc reduction of acutely elevated PPS, intended to alleviate attacks of angina pectoris. The preventive part included the following:

(1) Mandatory daily PPS measurements at home with instruction of how to perform PPS measurements, including a guideline for interpretation of the PPS measure, how to reflect on the measure, and a guide to clinical signs of alarm that require immediate attention.
(2) Mandatory daily cutaneous sensory nerve stimulation at specific sites on the body surface aimed at a reduction in elevated baseline PPS values and subsequent maintenance of low resting PPS.
(3) Daily recording of PPS measures in a web journal as a personal guide to the effect of the intervention, with ad hoc cognitive reflection in cases of sudden elevations of the PPS measure.
(4) Ongoing professional surveillance based on a personal web journal allowing pro-active professional intervention in cases of missing or deviating PPS measurements.
(5) A range of free-choice mental and physical exercises presented in the book of general stress management aimed at reducing stress in support of persistent lowering of resting PPS.

At the onset of active intervention, active group subjects learned by personal one-to-one instruction to identify tender spots on the chest bone (intended as a sign of an acutely elevated sympathetic activity); to apply moderate pressure with a finger at one of these locations, preferably the most tender one, without causing pain; and to maintain the pressure for 30–60 s until a reduction of the tenderness at the cutaneous pressure point. In participants with cases of angina pectoris, we expected to observe a concomitant subsidence of the angina pectoris attack. If not, we instructed the patient to take nitroglycerin. We interpret a marked reduction of tenderness at the cutaneous pressure point within the first minute of stimulation as evidence of correctly applied pressure, predicted to reduce elevated sympathetic activity. Without a reduction, the subject repeats the procedure at another tender skin surface point in the proximity. We instructed a spouse in daily cutaneous nerve stimulation at the back of the chest of the subject as a preventive measure, including ad hoc measures in cases of present angina. All participants received the information on how to conduct nerve stimulation on the back by themselves (e.g., using a small firm ball in a long stocking, with a knot on each side of the ball, for applying pressure against a wall) as an alternative or supplement to the nerve stimulation conducted by a spouse. Active intervention group members received a 40-page booklet with instructions into the program, as well as a quick guide card meant to always be available with general instructions on how to alleviate an attack of angina pectoris. After the three months of education, no further intervention-related instructions or intervention-related contact were provided. However, the participants were left with oral and written instructions on how to proceed with the intervention, when needed, including a contact card with instructions on how to manage chest pain.

### 2.7. Passive Intervention

Passive intervention group members continued the cardiac rehabilitation program initiated at least six months prior to the inclusion in the RCT. As the active group members, at the baseline examination, they received the information that their level of persistent stress was elevated as a sign of poor cardiovascular health, and they received the same 80-page manual of general stress with management suggested from the perspective that persistent stress negatively affects IHD. Thereafter, the passive group members received no further intervention-related instructions or intervention-related contact.

### 2.8. Statistics

The unique 10-digit Danish central person registry (CPR) number tracks all registered individuals with respect to mortality. We conducted three all-cause mortality analyses on an ITT basis [28] (for statistical details, see Supplementary Materials). We adopted the statistical methods from the two previous observational studies [22,23]: Statistics Denmark delivers all-cause mortality data for five-year intervals, allowing us to compare each of the 213 persons of the RCT to approximately 35,000 individuals with the same age and gender for the five-year observation period, each starting at the time of the entry into this study. We regarded the one-sided *t*-test as consistent with the test of the hypothesis of previously observed reductions of mortality by means of RCT that eliminates potential selection and researcher biases, but we present results of both one- and two-sided *t*-tests [29]. The primary analysis compared the all-cause mortality of the two groups of the RCT study with two corresponding subsets of the general Danish population, matched for age, gender, and observation period.

When comparing active and passive intervention groups of this RCT, we performed the analysis with time-dependent Poisson regression to estimate incidence rate ratios (IRRs, with 95% confidence intervals [CIs]) for the primary outcome of all-cause mortality. We adjusted the Poisson regression model for diabetes mellitus because of imperfectly balanced randomization at baseline, making diabetes more frequent in the active intervention group. We set up the Poisson model to follow individuals from time of inclusion and randomization of this RCT in the period 2011–2012 for a maximum of five years as predefined in the protocol. Individuals were censored at the time of death, or at the end of the follow-up period. We used a 5% significance level in all analyses including tests for interactions. We performed all analyses using SAS Statistical Software package version 9.4 (SAS Institute Inc., Gary, NC, USA) and Stata Software version 15 (StataCorp, College Station, TX, USA).

### 2.9. Meta-Analysis

In a separate analysis, we pooled mortality data from the active intervention group of the RCT with those of two previous observational studies [22,23]. As this RCT represents the third consecutive clinical study examining the effect of the same non-pharmacological treatment in patients with cardiovascular disease, we added the results of the of the two previous observational studies to this study using conventional statistics. The likelihood of obtaining a statistically significant result in three consecutive trials is the product of the following likelihood: (*p*total = *p*1 × *p*2 × *p*3). We compared each participant of the three active groups to approximately 35,000 individuals from the general population. We also completed a meta-analysis of the effect of the intervention on mortality justified by the two case–control studies and this RCT in which all patients had cardiovascular disease, underwent the same treatment analyzed by ITT, had the same all-cause mortality endpoint, and used the same control groups from the general Danish population matched for gender, age, and observation period. We performed this meta-analysis by pooling estimates from the three studies. We chose the random effects model, expecting the three included studies to be heterogeneous due to different designs. For each study, we used the reported estimate and its 95% confidence in this meta-analysis by pooling estimates by means of a generic inverse variance method, performing the meta-analysis with R version 4.2.3 (R Foundation for Statistical Computing, Vienna, Austria) from the R package “meta” version 6.2-0.

## 3. Results

We present demographic characteristics of the active and passive intervention RCT groups in Table 1. The prevalence of diabetes mellitus in the two RCT groups at baseline differed significantly, a difference considered in the statistical comparison of the mortality rates of the active and passive intervention groups of the RCT. However, we did not consider this difference when we compared mortality rates of each of the two RCTs with the corresponding control groups of the general population of Denmark. We observed no other significant between-group differences. During the five years of observation, we identified three or fewer deaths of subjects in the active intervention group, and eight deaths, including four heart-related and four non-heart-related deaths, in the passive intervention group (Table 2). Due to local regulations, we do not present the number of heart- and non-heart-related deaths in the active intervention RCT group of the subjects.

### 3.1. Active and Passive Intervention Groups versus General Population

**Figure 2:**
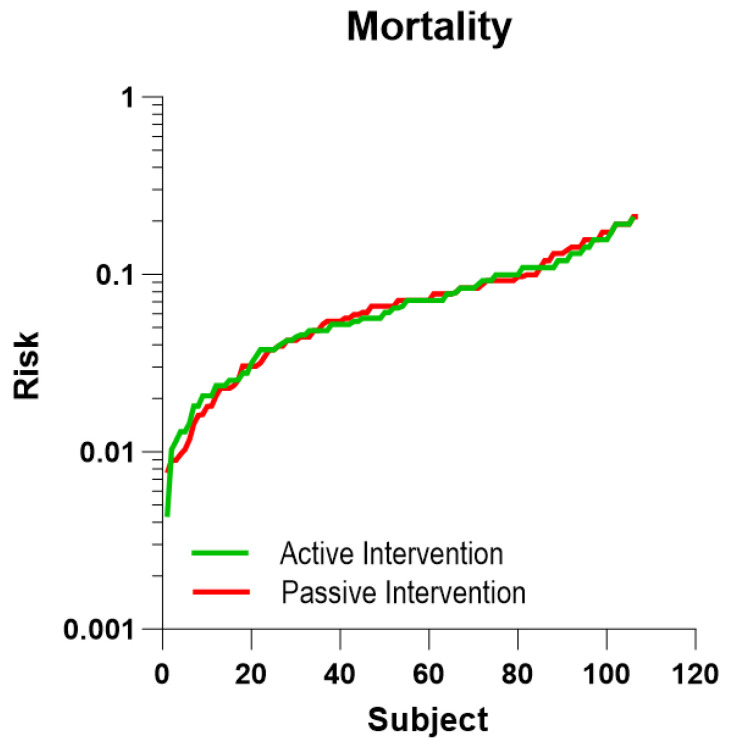
RCT populations. Distributions of individual 5-year risks of death for each subject of the RCT active intervention (N = 106) and passive intervention (N =107) groups. Each point represents one subject.

**Figure 3:**
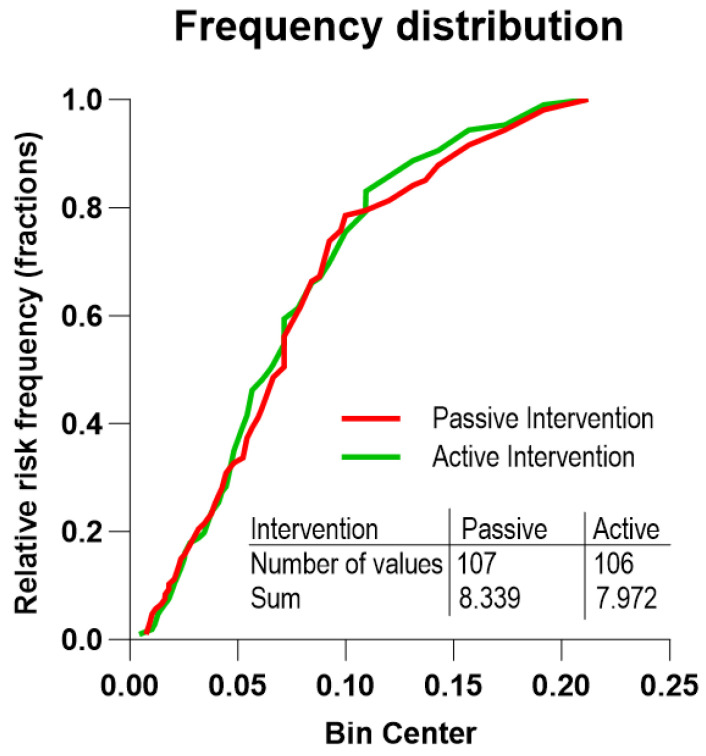
Predicted deaths from background population of Denmark. The accumulated number of expected 5-year deaths for the RCT active intervention and passive intervention groups, respectively (Y-axis). The abscissa (X-axis) (bin center) is the magnitude of the 5-year accumulated risks for the individual subjects, shown as groups of subjects within fractions (bins) of 0.05% risk of death within the 5-year observation period matched to the general Danish population with respect to gender, age, and observation period.

**Figure 4:**
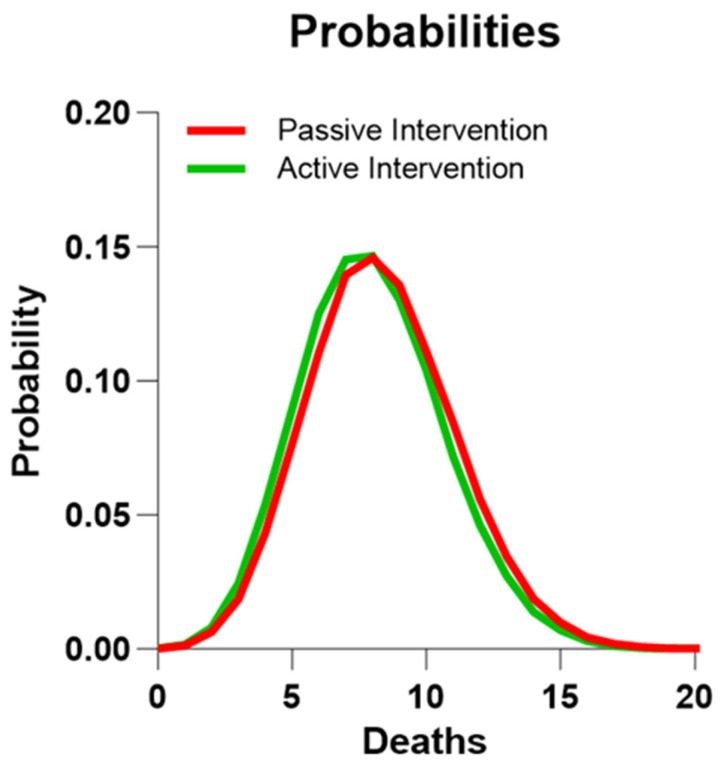
Predicted distributions of deaths in RCT groups. Distribution of probabilities of number of deaths during the 5-year observation period in two groups with regard to the RCT active intervention and passive intervention groups, respectively, when they are matched to the general Danish population for gender, age, and observation period.

The distribution of the individual five-year risks of death of the 106 subjects of the RCT active intervention group and the 107 subjects of the RCT passive group is non-symmetrical (Figure 2). Based on the background population of Denmark, we estimated the distribution of the number of deaths in two groups corresponding to the active and passive RCT groups, considering the individual risks. The two groups had predicted numbers of deaths of 7.97 and 8.34, respectively (Table 2, Figure 3 and Figure 4). Compared to the expected number of deaths during the five-year observation period, the number of three or fewer deaths of the active group significantly failed to reach the expected number of eight deaths (one-sided *t*-test, *p* = 0.010; two-sided *t*-test, *p* = 0.043), while the observation of eight deaths in the passive intervention group matched the prediction (*p* = 0.54).

### 3.2. Active versus Passive Intervention Groups

**Figure 5:**
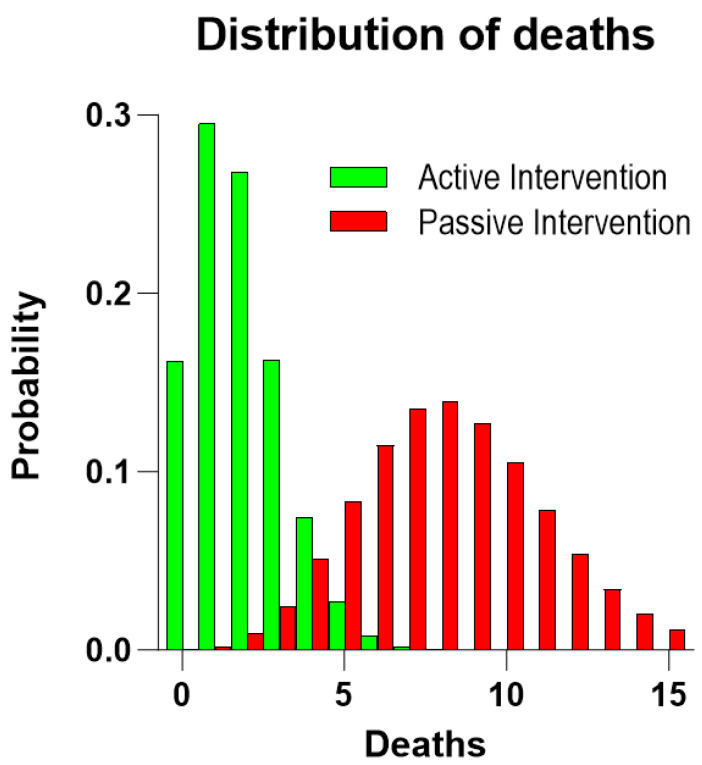
Distribution of probable deaths in RCT populations. The theoretical prediction of the distribution of the number of deaths in the RCT active intervention and passive intervention groups as incidence rates.

At baseline, 20 patients of the active group and 8 patients of the passive group had Type 2 diabetes mellitus (T2D) (between group *p* = 0.027). Adjusting for this difference, we found a significant difference of survival between the subjects of the active and passive intervention groups. During the five years of observation, we identified three or fewer deaths of subjects in the active intervention group, and eight deaths in the passive group. The all-cause mortality incidence rate was 3.1 per 1000 person-years for the active intervention group compared to an incidence rate of 12.5 per 1000 person-years for the passive intervention group (Figure 5). When we compared the active and passive intervention groups, the adjusted incidence rate ratio was 0.18 (95% confidence limits 0.04–0.90, two-sided *t*-test *p* = 0.035).

## 4. Discussion

**Figure 6:**
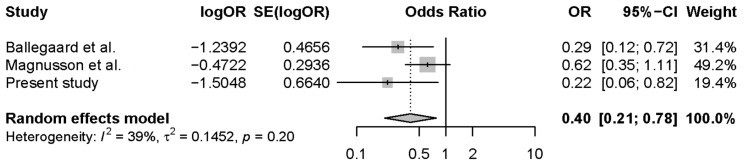
Meta-analysis with respect to 5-year all-cause mortality for three consecutive clinical trials in persons with a cardiovascular disease receiving a non-pharmacological intervention for PPS reduction and angina pectoris attack management (Ballegaard et al., 2004 [22]; Magnusson et al., 2010 [23].

In this RCT, we tested survival after a specific hypothetical adjunct intervention of IHD, claimed to ameliorate an assumed dysfunctional regulation of autonomic nervous system function with concomitantly elevated sympathetic activity, in association with pathologically elevated measures of PPS. We conducted this RCT as a single-center, two-armed, parallel-group, observer-blinded, randomized (1:1), clinical superiority trial. Compared to the general population, we found a substantial reduction in all-cause mortality, consistent with a hypothesis formulated from previous case–control studies. The present results demonstrated a 75% significant reduction of the relative risk of mortality in the active intervention group, compared to the general population. In contrast, we found no differences between the passive intervention group and the corresponding subsection of the general population. Compared to the passive intervention group of this RCT, the active RCT intervention group achieved a mean reduction in the relative all-cause mortality of 82%. The subsequent meta-analysis demonstrated a substantial 60% reduction in the 5-year all-cause mortality of members of the active intervention groups of individuals with a cardiovascular disease and elevated PPS, compared to the general population (Figure 6).

The active intervention group received three months of instruction into alleged reversal of ANSD and sympathetic hyperactivity with PPS reduction, and in angina attack management. The participants all had stable IHD, with at least one episode of MI, or an intervention with PCI or CABG, more than a year before entering this study. As depicted in the demographics Table 1, more than 40% of the participants reported a Canadian Cardiovascular Society (CCS) angina score class of 1 or more, and 34% reported resting angina. Thirty-six percent received diuretics, primarily due to a history of heart failure. Frequent contacts with health care providers were recorded. In clinical practice, participants were symptomatic and represented a population at moderate risk of new ischemic events, and the 8% observed 5-year all-cause mortality of the control group matches the results of a recent review finding an equal 5.7-year all-cause mortality for conservative (8.5%) and invasive treatment (8.2%) [30].

### 4.1. PPS Measure, ANSD, and Mortality of Patients with Ischemic Heart Disease

Despite substantial improvements of the survival in patients with IHD, patients still face an elevated risk compared to the general population [31], particularly in cases of concomitant T2D [32]. Given the patients with IHD of this RCT of whom a substantial number had T2D as well, it is of interest that we found the mortality of the active intervention group to be significantly lower than that of the general Danish population.

In earlier studies with the RCT design [33,34], the active intervention reduced elevated PPS measures, which is associated with a concomitant reductions of a range of independent health risk factors known to affect mortality in association with the apparent changes of ANS function. Thus, in healthy individuals, measures of blood pressure, heart rate, work of the heart measured as the pressure rate product, plasma lipids, and glycated hemoglobin all declined in response to the intervention [33]. Also, in T2D, measures of glycated hemoglobin [34] and evidence of disturbed homeostatic regulation of glucose metabolism both declined in response to the intervention [35].

Medications that block beta-adrenergic activity are effective as inhibitors of elevated sympathetic activity in patients with IHD, but this use was recently questioned with respect to the risk/benefit ratio, due to a broad range of significant side effects [36]. We have repeatedly demonstrated that while PPS was unaffected by this medication, the effects from reducing an elevated PPS on depression, heart rate variability and tilt table response were blocked [34], suggesting that the mechanism of this intervention is a centrally mediated reduction of elevated sympathetic activity, and with little risk of side effects.

On this basis, we tested the claim that a specific, adjunct, non-pharmacological intervention, associated with hypothetical reductions of ANSD and concomitantly elevated sympathetic activity, linked to a decline in PPS, is a possible cause of reduced mortality of subjects of the active intervention. Searching for other causes, we found no evidence that a reduction of other cardiovascular risk factors is associated with lower all-cause mortality of the magnitude revealed by the present findings [31]. Confounding factors that may affect IHD mortality include changes in smoking habit, physical exercise, diet, body weight, yoga, mindfulness, and other psychological interventions including cognitive therapy, religion, and spirituality. However, none of these have been shown to reduce mortality to the level of the present findings or below that of the general population [37,38,39,40,41,42,43,44,45,46].

### 4.2. Modulation of Sensitivity to Periosteal Pressure

We define neuromodulation as mechanisms of chemical, electrical, or mechanical pain induction, as well as of autonomic sympathetic activity [47] that generate afferent impulses to the brain. Although the methods of sensory nervous stimulation are different, it is possible that the same afferent signals reach the central ANS for different purposes, depending on the specifics of the stimulation of nervous tissue. Neuronal stimulations include vagal or sacral nerve stimulations [48,49,50], spinal cord stimulation [51,52], and non-noxious cutaneous sensory nerve stimulation [53,54]. Non-noxious sensory nervous stimulation is known to reduce diabetic neuropathy [51] and to reduce the number of angina pectoris attacks in patients with IHD [52]. The effect is reminiscent of the well-known autonomic reflex arch regulating pain sensation in the shape of diffuse noxious inhibitory control by reducing the efferent response [55,56]. Neuromodulation with non-noxious cutaneous sensory nervous stimulation is a well-known and widely used treatment modality for newborn infants, and with a substantial increase in overall survival [17]. The treatment contributes by reducing stress [18]. Non-noxious cutaneous sensory stimulation is a key feature of the active intervention used in this study that includes moderate pressure stimulation at the back of the patient performed twice daily by spouse or partner, a feature that is identical to the skin-to-skin treatment for infants. For these reasons, we suggest that future experimental testing of a hypothetical regulation of the central nervous system with PPS is of interest.

### 4.3. Meta-Analysis of Pooled Data

We registered the pooled active intervention mortality of 1168 person-years, calculated as 103 persons followed for 3 years of observation [22], plus 73 persons followed for 4.5 years of observation [23], plus 106 persons followed for 5 years of observation in this study. The comparison reached a total number of person-years of approximately 40 million person-years from the pooled general Danish population, calculated as 1168 person-years of the active group times the 35,000 persons of the general population that match each of the 1,168 persons. The likelihood of obtaining a statistically significant result in three consecutive trials is the product of the likelihoods (*p*total = *p*1 × *p*2 × *p*3; in this pooled analysis: *p* < 0.05 × *p* < 0.1 × *p* = 0.01: *p*-total < 0.00005). We pooled the estimates from two previous observational studies and this study in a random effects meta-analysis. The pooled estimate showed a significantly lower mortality (OR: 0.40; 95% CI: 0.21–0.78, *p* = 0.007), albeit with some insignificant heterogeneity (I^2^ = 0.39, *p* = 0.20) (Figure 6).

### 4.4. Strengths and Limitations

This study has several strengths, including access to robust five-year mortality data from Statistics Denmark. We compared the alleged effect on mortality of the active intervention in patients with IHD to the mortality of the general population, matched for gender, age, and observation periods, thus allowing comparison with a control group of approximately 19 million person-years. The statistics revealed that the two control groups of the general population with respect to the active and passive intervention RCT groups had identical predictions of mortality. Furthermore, the matched observed and predicted mortality records of the passive intervention group of individuals were similar in magnitude to patients receiving a conservative or invasive intervention [30].

In contrast to the general challenge of low adherence to traditional non-pharmacological cardiac rehabilitation programs [20,21], the special features of the present active intervention program aimed at personal adherence and compliance identified a persistently high compliance rate above 80% in healthy persons [33], in women with breast cancer [57], in individuals with IHD [25], and in patients with T2D [34]. We considered this evidence from multiple studies as a strength with respect to the daily adherence to the present cardiac rehabilitation program.

This RCT is the ultimate test of the hypothesis generated by two previous long-term observational studies in patients with IHD or stroke. The previous studies add strength to the present findings because of identical interventions, identical control groups (i.e., the general population), identical statistical analyses (i.e., ITT analyses), and robust reductions in mortality in patients in the active intervention in all three studies, with the risk of selection or researcher bias eliminated in the RCT. As such, the elimination formed the basis for this meta-analysis designed to reduce the risk of type 1 statistical error by increasing the number of events. Accordingly, this meta-analysis was conducted after the results of the RCT were obtained.

As in the previous hypothesis-generating studies, ITT analysis in this RCT is the best possible test of the evaluation of mortality [28]. The ITT analysis reflects the compliance when implemented. In an earlier study of the same study population, we used ITT analysis together with per protocol (PP) analysis when evaluating the 3-month effect on depression symptoms and PPS (see also CONSORT diagram), with no significant difference between the two analyses [25]. Further, both analyses demonstrated a large effect size (i.e., Cohen effect size > 0.7 [58]) with respect to a reduction in PPS. In this study, we did not complete PP analysis because it would be unclear how to record compliance during 5 years of no observation after the initial 3 months. Further, PP analysis would introduce potential bias from the between-group comparability, as obtained with the randomization procedure, as well as from treatment-confounding feedback loops [59].

It is a limitation that we observed only a small number of events in this RCT. We expected the statistical tests of the effect to have low power and to reflect a high risk of a type 1 error. To address this limitation, we tested the effect of intervention on all-cause mortality in the following two ways: by comparison to the general population and by comparison of the two RCT groups. We reduced the risk of type 1 statistical errors by conducting this RCT as a culmination of the two previous prospective case–control studies and performance of a meta-analysis. Furthermore, the findings made sense from a pathophysiological point of view, as previous studies have demonstrated a relevant effect from reducing elevated PPS on a broad range of cardiovascular health risk factors known to be associated with ANSD and to affect mortality [25,26,33].

Considering a hypothetical 50% increase in the number of deaths in the active intervention RCT group, the reduction in all-cause mortality remained significant compared to the general Danish population, matched for age, gender, and observation period.

The observed 80% relative reduction in all-cause mortality in the active group of this RCT is a surprise. For new interventions within recent decades, the reduction rarely exceeded 25%, probably reflecting that each of these interventions approached one health risk factor, only [60]. This suggests that a reduction in an elevated PPS measure may reflect a more global impact on health, affecting a broad range of health risk factors at the same time, hypothetically through the regulation of ANS function in the brain [33].

In this context, it is worth noting that the use of beta-blockers in patients with IHD inhibits the efferent and peripheral sympathetic activity of ANS function but does not affect the PPS measure. This might occur as PPS is regulated centrally in areas of the brain with no beta-adrenergic receptors. The only area in the brain which does not have these receptors seems to be the orexin receptor system in the lateral hypothalamus. This anatomical site also seems to regulate ANS function, including stress, and warning and defense systems [34]. Further studies are warranted to explore such a possibility and may include functional brain scanning and use of orexin receptor antagonists.

It is a potential weakness that we had no contact with the participants after the three months after the initial intervention. However, we chose this design deliberately to avoid bias from a non-specific “tender, love and care effect” present in participant–researcher interactions. The intervention is educational, and we left the active intervention group members with oral and written instructions on how to proceed with the intervention, when needed, including a contact card with instructions on how to manage chest pain. Exclusion of participant contacts contrasts with conventional scientific testing of a cardiac rehabilitation program. Together with two other factors, the use of ITT analysis and the use of the general population as the control group (rather than individuals with established IHD) establishes the toughest possible conditions for the testing of the hypothesis.

## 5. Conclusions

A considerable reduction in 5-year all-cause mortality in individuals with stable IHD who underwent a 3-month intervention program was reported, and with substantial statistical significance. The present results of this RCT are consistent with results from previous case–control studies in individuals with IHD or stroke subjected to the same educational intervention. The consistency adds evidence to the benefits of effects from the used intervention aiming at and accompanied by a PPS reduction with respect to a variety of cardiovascular risk factors alleged to be controlled by the ANS. As such, the proposed intervention that focused on autonomic dysfunction and IHD is potentially applicable as a supplement to conventional treatment. The PPS tool and the associated treatment are applicable for both diagnostic, therapeutic, and preventive purposes, with little risk of side effects.

## Figures and Tables

**Table 1:** Distribution of baseline factors according to RCT treatment groups.

	Full Sample	Active Group	Passive Group	*p*-Value
Number of participants (n)	213	106	107	
Male (n, %)	156 (73%)	78 (74%)	78 (73%)	NS *
Age in years (mean, SD)	62 (8.1)	62 (8.1)	62 (8.2)	NS
*Psychometrics*				
MDI (mean, SD)	8.9 (7.4)	8.4 (7.7)	9.4 (7.0)	NS
WHO-5 (mean, SD)	65 (19)	67 (19)	63 (19)	NS
PPS (mean, SD)	81 (13)	81 (13)	81 (13)	NS
SF-36 PCS (mean, SD)	48 (8.4)	48 (9.1)	48 (7.6)	NS
SF-36 MCS (mean, SD)	52 (9.3)	53 (9.3)	52 (9.3)	NS
CSS (mean, SD)	9.7 (7.1)	9.2 (6.5)	10 (7.6)	NS
*Social status*				
Married or cohabitating (n, %)	175 (82%)	83 (78%)	92 (86%)	NS
Have children (n, %)	190 (92%)	97 (91%)	96 (90%)	NS
*Employment status*				
Employed (n, %)	106 (50%)	54 (51%)	52 (49%)	NS
Unemployed (n, %)	4 (2%)	3 (3%)	1 (1%)	NS
Retired (n, %)	92 (47%)	46 (44%)	52 (48%)	NS
*Cardiac variables*				
Self-reported time (years) since first AMI (mean, SD)	7.5 (5.8)	8.2 (6.5)	6.8 (5.0)	NS
Previous AMI (n, %)	136 (64%)	69 (65%)	67 (63%)	NS
Treated with PCI (n, %)	147 (69%)	73 (69%)	74 (69%)	NS
Treated with CABG (n, %)	52 (24%)	27 (25%)	25 (23%)	NS
In-hospital days during last 12 months before inclusion due to cardiac disease (n, (%), mean number of days)	46 (22%) 6 days	18 (17%) 6 days	28 (26%) 7 days	NS
Visits to cardiac outpatient clinic during last 12 months before inclusion (n (%), mean number of visits	68 (32%), 3 visits	27 (25%) 4 visits	41 (39%) 3 visits	NS
Visits to cardiologist during last 12 months before inclusion (n (%), mean number of visits	29 (16%) 2 visits	13 (12%) 2 visits	16 (15%) 2 visits	NS
Visits to general practitioner during last 12 months before inclusion for cardiac disease (n (%), mean number of visits	90 (47%) 3 visits	46 (43%) 3 visits	44 (42%) 2 visits	NS
CCS Angina Pectoris Class I	55 (26%)	26 (25%)	29 (27%)	NS
CCS Angina Pectoris Class II	25 (12%)	14 (14%)	11 (10%)	NS
CCS Angina Pectoris Class III	5 (2%)	1 (1%)	4 (4%)	NS
CCS Angina Pectoris Class IV	2 (1%)	1 (1%)	1 (1%)	NS
Chest pain at rest	71 (34%)	39 (38%)	32 (30%)	NS
Resting pulse (mean, SD)	61 (11)	61 (11)	60 (11)	NS
MAP (mean, SD)	98 (10)	98 (9.7)	97 (11)	NS
*Cardiac risk factors*				
BMI (mean, SD)	27.6 (4.3)	27.8 (4.3)	27.4 (4.4)	NS
Triglyceride (mean, SD)	1.5 (0.9)	1.4 (0.7)	1.5 (1.0)	NS
Current smoker (n, %)	22 (10%)	9 (9%)	13 (12%)	NS
Heart rate ≥ 70 beats/minute (n, %)	35 (16%)	20 (19%)	15 (14%)	NS
*Self-reported co-morbidity*				
Heart failure (n, %)	72 (34%)	29 (27%)	43 (40%)	NS
Chronic obstructive lung disease (n, %)	13 (6%)	5 (5%)	8 (8%)	NS
Diabetes (n, %)	28 (13%)	20 (19%)	8 (8%)	*p* = 0.015
Previous cerebral insults (n, %)	15 (7%)	7 (7%)	8 (8%)	NS
Have been treated for depression (n, %)	32 (15%)	12 (11%)	20 (19%)	NS
Elevated depression score (i.e., MDI score ≥ 15, indication incipient depression)	41 (19%)	21 (20%)	20 (19%)	NS
*Medication*				
Beta-blockers (n, %)	125 (60%)	65 (61%)	60 (57%)	NS
Cholesterol-lowering medication (n, %)	188 (90%)	94 (89%)	94 (88%)	NS
Calcium antagonists (n, %)	47 (23%)	26 (25%)	21 (20%)	NS
Angiotensin-II antagonist and/or ACE inhibitors (n, %)	115 (55%)	56 (53%)	59 (55%)	NS
Diuretics (thiazide or furosemide) (n, %)	74 (36%)	40 (39%)	34 (33%)	NS
Anti-depressive medication (n, %)	12 (6%)	4 (4%)	8 (8%)	NS

* NS: *p* > 0.05 between active group and passive; BMI: body mass index; MDI: major depressive inventory; WHO-5: quality of life questionnaire; SF-36 PCS: short-form self-report health questionnaire physical component summary; SF-36 MCS: short-form self-report health questionnaire mental component summary; CSS: clinical stress symptom score questionnaire; AMI: acute myocardial infarction; PCI: percutaneous coronary intervention; CABG: coronary artery bypass grafting; MAP: mean arterial blood pressure; CCS Angina Pectoris Class: Canadian Cardiovascular Society angina score; ACE: Angiotensin Converting Enzyme.

**Table 2:** Distribution of deaths.

RCT Group	n	Five-Year Risks of Death Mean (Range)	Predicted Number of Deaths	Number of Deaths (95% CI)	*p*-Value
Active group	106	0.075 (0.0043–0.2123)	7.97	≤3 (0.5–6.6)	0.01
Passive group	107	0.078 (0.0076–0.2123)	8.33	8 (3.8–14.1)	NS

Distribution of five-year risks of death (mean (range)), predicted number of deaths in the active and passive RCT intervention groups (when matched to general Danish populations for age, gender, and observation period), and observed number of deaths (CI = confidence limits; NS: = non-significant).

## Data Availability

The data underlying this article will be shared upon reasonable request to the corresponding author.

## Supplementary Materials

The following supporting information can be downloaded at: https://www.mdpi.com/article/10.3390/jcm12247585/s1, Figure S1: Using Monte Carlo technique, the figure shows the distribution of number of deaths in normal populations who matches the gender, age and calendar year of the active intervention and passive intervention group (control) of the RCT, respectively.; Table S1: Probabilities of extreme number of deaths in the active intervention and passive intervention groups of the RCT, matched to the general Danish population matched for gender, age and observation period [61,62,63].

## Abbreviations

ANS	Autonomic nervous system
ANSD	Autonomic nervous system dysfunction
CCS	Canadian Cardiovascular Society grading of angina pectoris
CONSORT	Consolidated Standards of Reporting Trials
IHD	Ischemic heart disease
ITT	Intention-to-treat
PP	Per protocol
PPS	Periosteal pressure sensitivity
T2D	Type 2 diabetes mellitus
