# Supplementary material for: In Ischemic Heart Disease, Reduced Sensitivity to Pressure at the Sternum Accompanies Lower Mortality after Five Years: Evidence from a Randomized Controlled Trial
Source: J Clin Med. 2023 Dec 8;12(24):7585. doi: 10.3390/jcm12247585 (PMC10744062; doi:10.3390/jcm12247585)
Supplement: Supplementary file 1 [file jcm-12-07585-s001.zip › jcm-2690384-supplementary.pdf]

Supplementary Materials

In Ischemic Heart Disease, Reduced Sensitivity to Pressure at the Sternum Accompanies Lower Mortality after Five Years: Evidence from a Randomized Controlled Trial

Søren Ballegaard [1,*,†], Jens Faber [1,2,†], Christian Selmer [1,2,3], Finn Gyntelberg [4], Svend Kreiner [5], Benny Karpatschof [6], Tobias Wirenfeldt Klausen [1], Ake Hjalmarson [7] and Albert Gjedde [2,8,9,10]

[1] Endocrine Unit, Department of Medicine, Herlev-Gentofte University Hospitals, 2730 Herlev, Denmark; jens.faber@regionh.dk (J.F.); christian.selmer@regionh.dk (C.S.); tobias.wirenfeldt.klausen@regionh.dk (T.W.K.)
[2] Faculty of Health and Medical Sciences, University of Copenhagen, 2200 Copenhagen, Denmark; gjedde@sund.ku.dk
[3] Department of Endocrinology, Bispebjerg-Frederiksberg University Hospitals, 2400 Copenhagen, Denmark
[4] The National Research Center for the Working Environment, 2100 Copenhagen, Denmark; finn.gyntelberg@gmail.com
[5] Institute of Biostatistics, University of Copenhagen, 1017 Copenhagen, Denmark; svend.kreiner@mail.tele.dk
[6] Institute of Psychology, University of Copenhagen, 1017 Copenhagen, Denmark; benny@karpatschof.dk
[7] Department of Cardiology, Sahlgrenska University Hospital, University of Gothenburg, 41345 Gothenburg, Sweden; ake@cardwijk.se
[8] Department of Neuroscience, University of Copenhagen, 2200 Copenhagen, Denmark
[9] Translational Neuropsychiatry Unit, Department of Clinical Medicine, Aarhus University, 8000 Aarhus, Denmark
[10] Department of Neurology and Neurosurgery, McGill University, Montreal, QC H3A 1A1, Canada
* Correspondence: soeren.ballegaard@regionh.dk
† These authors contributed equally to this work.

Supplementary Data on Statistics

Methods

Statistics

Comparing mortality to that of the general Danish population

For the comparison of mortality between the two groups of the RCT and that of the general Danish population, we estimated the distribution of deaths in the subgroup of persons from the Danish general population, based on 5-year all-cause mortality data from Statistics Denmark, with the same age-and sex- profile as each individual patient of the initial randomized trial, and using a 5-year observation, which started at the initiation of the RCT (25). This means that each patient of that study was compared to approximately 35.000 persons.

As the distribution of the induvial five-year risk of death of the 106 persons of the active intervention group are discrete and non-symmetrical, we estimated the distributions of the number of deaths, taking into account the individual risks. And therefore, we also estimated the distribution of death for the active intervention and passive intervention group members, respectively, by Monte Carlo methods (61), by counting the number of deaths in very a large number of simulated samples where the probabilities of events are defined by the five-year risks associated with the 213 individual persons of the initial randomized trial. The results of such procedures are known to be unbiased and with a standard error defined by the number of Monte Carlo samples. In the current study, the distributions of the probabilities in the normal population matched to the active and passive intervention groups of the RCT were estimated by 100.000 Monte Carlo samples. Supplementary Figure 1 shows the expected number of deaths during the 5-year observation period in the normal population matched to

the active intervention and passive intervention group of the RCT, respectively. The Monto Carlo calculations confirmed the estimated death contributions from Statistics Denmark.

As the distribution of deaths is discrete, we did not define critical areas with the exact p-values of 5% risk of a Type 1 error but calculated the probability of death with critical areas with sizes close to p-values of 5 %, e.g., 0-3 number of deaths and more that 15 deaths (Supplementary Table 1). The table shows these data for active intervention and passive intervention group of the RCT, respectively, and shows that there is no significant difference between the two groups, and thus shows that the calculated control group of the RCT from the general population, represents a reliable control group with respect to all-cause mortality statistics concerning test of the hypothesis, that the active intervention reduces all-cause mortality, when compared to the general population. Furthermore, it shows that the probability of the observed number of deaths in the active intervention group (presented as three or less deaths according to local regulations), compared to the general population is significant; one side P = 0.1003) (two-sided P= 0.043), and thus that the the hypothesis of a reduced mortality by the active intervention is rejected by two-sided statistics as well.

Comparing mortality of the two groups of the RCT study

We compared mortality of the active and passive intervention groups of members of the RCT, including all participants in the original study on an intention-to-treat basis, followed by access to nationwide registries with the primary outcome selected as all-cause mortality. For the comparison of the mortality between the active and control groups of participants in the RCT, survival analysis used Poisson regression of rare outcome events with the assumption of constant risk of death in the relevant time-intervals (62,63), confirmed by sensitivity analysis of shorter time intervals. Using a time-dependent Poisson regression model, we estimated the incidence rate-ratio with 95% confidence intervals for the primary outcome. Study participants were censored at the time of death, the end of the follow-up period (31 December 2016), or at migration.

Supplementary Table

Table S1. Probabilities of extreme number of deaths in the active intervention and passive intervention groups of the RCT, matched to the general Danish population matched for gender, age and observation period.

Number of deaths	Active intervention	Control
0	0.028 %	0.011 %
1	0.168 %	0.135 %
2	0.807 %	0.635 %
3	2.443 %	1.855 %
0 – 3	3.046 %	2.636 %
15	0.696 %	0.994 %
16	0.290 %	0.432 %

17	0.115 %	0.194 %
18	0.049 %	0.082 %
19	0.016 %	0.021 %
15 – 106/107	1.266 %	1.742 %
0-3 or 15-106/107	4.312 %	4.378 %

Supplementary Figure

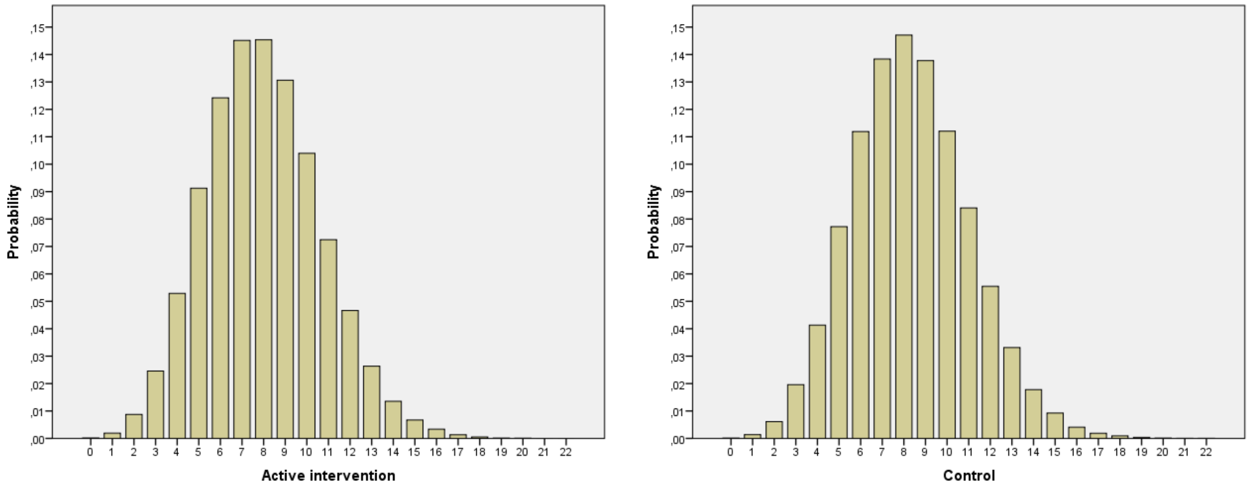

‘

Figure S1. Using Monte Carlo technique, the figure shows the distribution of number of deaths in normal populations who matches the gender, age and calendar year of the active intervention and passive intervention group (control) of the RCT, respectively.
